# The Y chromosome of autochthonous Basque populations and the Bronze Age replacement

**DOI:** 10.1038/s41598-021-84915-1

**Published:** 2021-03-10

**Authors:** Javier Rodriguez Luis, Leire Palencia-Madrid, Vivian C. Mendoza, Ralph Garcia-Bertrand, Marian M. de Pancorbo, Rene J. Herrera

**Affiliations:** 1 Area de Antropología, Facultad de Biología, Universidad de Santiago de Compostela, Campus Sur s/n, 15782 Santiago de Compostela, Spain; 2 BIOMICs Research Group, Dpto. Z. y Biologia Celular A., Lascaray Research Centre, University of the Basque Country UPV/EHU, Vitoria-Gasteiz, Spain; 3 Department of Molecular Biology, Colorado College, Colorado Springs, CO 80903 USA

**Keywords:** Evolution, Genetics

## Abstract

Here we report on the Y haplogroup and Y-STR diversity of the three autochthonous Basque populations of Alava (n = 54), Guipuzcoa (n = 30) and Vizcaya (n = 61). The same samples genotyped for Y-chromosome SNPs were typed for 17 Y-STR loci (DYS19, DYS385a/b, DYS398I/II, DYS390, DYS391, DYS392, DYS393, DYS437, DYS438, DYS439, DYS448, DYS456, DYS458, DYS635, Y-GATA H4) using the AmpFlSTR Yfiler system. Six major haplogroups (R, I, E, J, G, and DE) were detected, being R-S116 (P312) haplogroup the most abundant at 75.0% in Alava, 86.7% in Guipuzcoa and 87.3% in Vizcaya. Age estimates for the R-S116 mutation in the Basque Country are 3975 ± 303, 3680 ± 345 and 4553 ± 285 years for Alava, Guipuzcoa and Vizcaya, respectively. Pairwise *Rst* genetic distances demonstrated close Y-chromosome affinities among the three autochthonous Basque populations and between them and the male population of Ireland and Gascony. In a MDS plot, the population of Ireland segregates within the Basque cluster and closest to the population of Guipuzcoa, which plots closer to Ireland than to any of the other Basque populations. Overall, the results support the notion that during the Bronze Age a dispersal of individuals carrying the R-S116 mutation reached the Basque Country replacing the Paleolithic/Neolithic Y chromosome of the region.

## Introduction

Traditionally, Basque populations have been considered one of the oldest human isolates and direct descendant of upper Paleolithic (38,000–10,000 years) groups. They are resident of the Franco-Cantabrian region since the Late Glacial and Postglacial periods^1^. In Spain, Basque communities are distributed among narrow valleys within mountainous terrains in the north central region of the Iberian Peninsula. This geography may have had an effect on the Basques’ unique cultural, linguistic and genetic characteristics.

Over the years studies have examined the Basques using linguistic, archaeological, anatomical and genetic markers^2^. These include studies based on blood groups, serum proteins and enzymes^3,4^, mini-satellites^5^, autosomal microsatellites^6^, Y-specific microsatellites^7–10^, Y-specific single nucleotide polymorphisms^11,12^, mitochondrial DNA (mtDNA)^13^, polymorphic *Alu* insertions (PAIs)^14^, and high-density SNPs^15^. A number of studies using various types of genetic markers including HLA^16^, mtDNA^17,18^, microsatellites^19^, minisatellites^5^, Y Chromosome-specific^20,21^, autosomal SNPs^22^ and high density SNPs^23^ have contested the uniqueness of the Basques claiming that the Basques fall well within the European genetic spectrum.

A number of genetic markers have clearly shown an underlying genetic similarity shared among Basque groups. These include HLA antigens^24^ and SNPs^25^. Furthermore, some data based on classical markers^3^ and HLA studies demonstrate a lack of subpopulation structure among the Basque^16^. On the other hand, other studies based on classical and autosomal markers^26–29^ indicate genetic heterogeneity among Basque populations. Furthermore, investigations using classical markers^30–32^, immunoglobin allotypes^33,34^ and mtDNA sequence comparisons^35^ illustrate variable levels of substructure among the Basque. In a comprehensive study of uniparental markers in Basques and neighboring Indo-European-speaking populations from the Franco-Cantabrian region of Europe, it was concluded that the Basque-speaking populations fall within the genetic spectrum of other Western European populations^36^. Furthermore, the study suggests that any genetic heterogeneity and substructure observed in the Basque today relative to geography result from pre-Roman tribal structure dating back to the Bronze Age.

Recently, high-coverage genome-wide SNP data utilizing ancient DNA from Iberian samples from the past 8000 years confirms the genetic isolation of the Basques since the Bronze Age, ~ 2200–900 BCE^37^. The study describes the Basques as an Iron Age population genetically impacted by migrations from the Pontic-Caspian STEPPES. These population movements into Iberia likely correlated with the introduction of the Urnfield tradition and Indo-European languages to the region^38^. Yet, in Iberia only the Basque groups failed to adopt an Indo-European language. According to the study, these STEPPE migrants initially co-existed with the local Neolithic farming communities and eventually mixed to generate the Bronze Age Iberian population about 4000 years ago. On the average, the current Iberian populations possess about 40% Pontic-Caspian DNA^39^. In some Iberian communities the native Y-chromosomes of Neolithic farming communities were almost totally replaced by the Indo-European R1b lineages^39^. For unknown reasons, the replacement of Copper Age Western European Y chromosomes made up mainly of haplogroups I2, G2, and H by R1b Bronze Age chromosomes was more dramatic in Iberia. This sex-specific replacement suggests a higher contribution of incoming males than females, which is also supported by a lower X-chromosome input from the STEPPES. Today this Y chromosome turnover is particularly pronounced in the Basques, which exhibit 87% R1b^19^. In the rest of Iberia the abundance ranges from 43% in Malaga to 81% in Catalonia^40^. The population-dynamic mechanisms that generated such a sex-specific replacement are unknown.

According to Myers et al.^41^ the M269 mutation that defines Y haplogroup R1b originated in the Near East and travelled with Neolithic farmers to Northern Anatolia and then to the Pontic-Caspian steppe where a number of its subclades became closely associated with the spread of Indo-European languages into Western Europe. A subsequent mutation, S116 (also known as P312), likely occurred in what is today France approximately 5500–5000 years ago^41^. The R1b Y-chromosome haplogroup in Iberia is mainly represented by the R-S116 haplogroup, which reaches 80% in the Basque Country (average of all Basque Provinces)^42^. A derivative lineage of S116, DF27, which likely originated in Iberia reaches a maximum value of 63% in the Basque Country and minimum value of 40% in Galicia^12^.

Considering the elevated level of Indo-European Y chromosomes observed in the general Basque population, while retaining mtDNA and autosomal Neolithic sequences, we decided to investigate the Y-chromosome constitution of the three regional autochthonous Basque populations of Alava, Guipuzcoa and Vizcaya. In doing so, we were testing the hypothesis that Paleolithic/Neolithic Y chromosomes were replaced by S116 Y chromosomes in the three populations during the Bronze Age.

## Materials and methods

### Sample collection and DNA isolation

**Figure 1:**
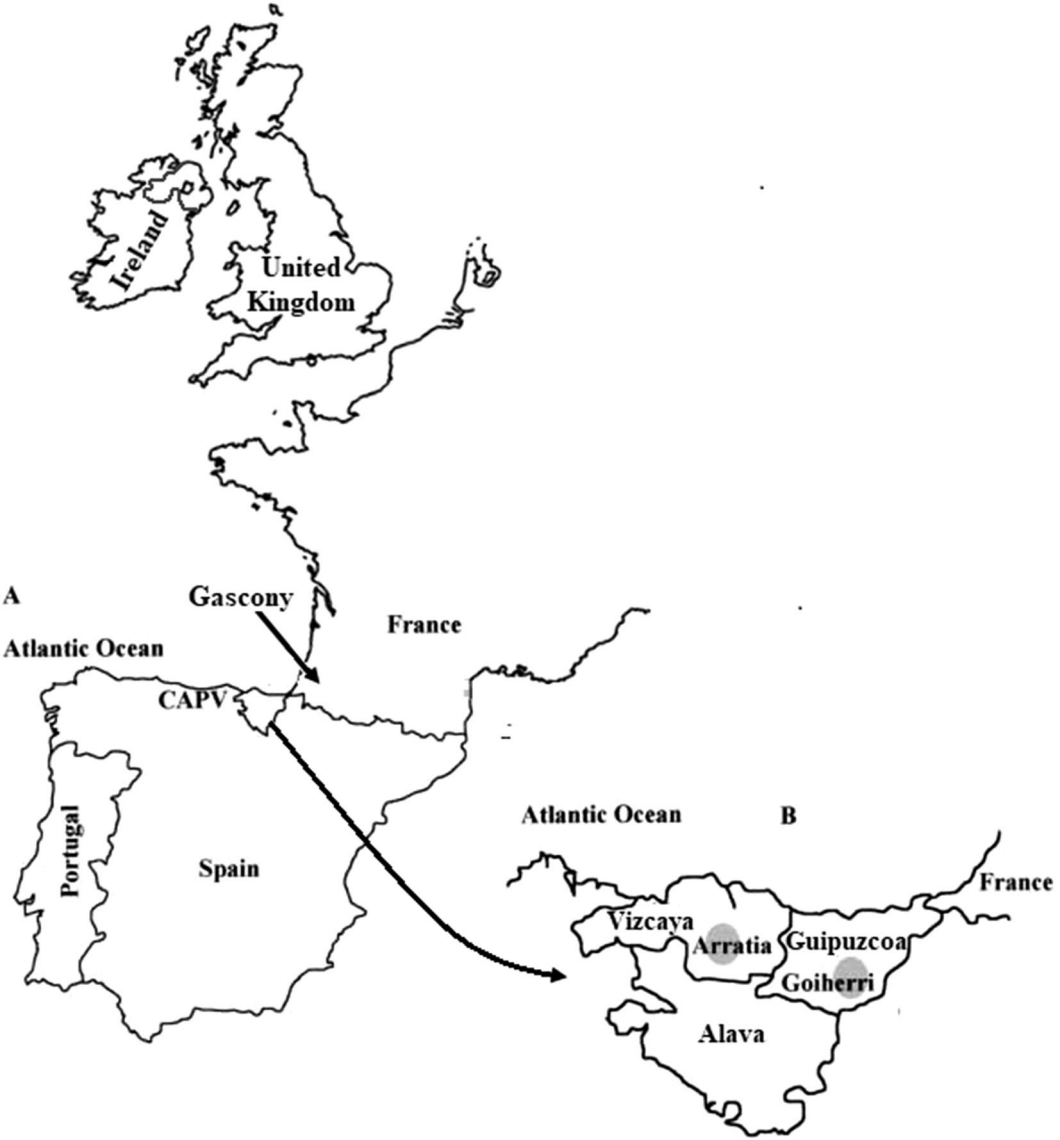
Basque Country and Western Europe.

Whole blood samples were collected from 145 autochthonous Basque individuals from the provinces of Guipuzcoa (n = 30), Vizcaya (n = 61) and Alava (n = 54). Peripheral blood was collected in EDTA vacutainer tubes by venipuncture from unrelated healthy individuals. All samples were procured from donors voluntarily while closely adhering to the ethical guidelines stipulated by Colorado College, Colorado Springs, Colorado USA and the University of the Basque Country, UPV/EHU, Vitoria-Gasteiz, Spain. All donors gave their informed consent prior to inclusion in the study, following the ethical principles and guidelines of the Declaration of Helsinki for the protection of human subjects. DNA was extracted from leukocytes by the phenol–chloroform method described by Batzer and Deiniger^43^. Individuals were considered autochthonous Basque if the four grandparents were born in the Arratia (Vizcaya province) and Goiherri (Guipuzcoa province) valleys of the Basque Country as indicated in Fig. 1. In the case of the Alava sample, the criterion was that the four grandparents were born in the Alava province.

### Reference populations

A total of 30 geographically targeted key reference populations with Y-STR or Y-SNP frequency data were included for phylogenetic comparisons to the three Basque populations. The reference populations were strategically selected providing phylogenetic context to the Basques populations genotyped. All collections, their abbreviations, biogeographical origin, reference publication and total number of individuals analyzed are listed in Supplementary Table 1.

### Y-chromosome SNP genotyping

A total of 49 bi-allelic markers (YAP (*Alu*), M35, M78, V65, M281, V12, V13, V22, V6, P72, M123, M107, M293, M183, M81, M201, P287, M377, P15, M286, P16, M258, M253, M21, L22, P109, M227, M26, P259, M304, P58, L24, M158, M9, M207, M198, M269, M412, U106, S116, U152, M126, M160, M529, M222, M65, M153, M167, M20) were examined using standard methods including PCR–RFLP^44^, allele-specific PCR^45,46^, PCR amplification and electrophoretic detection of Y-chromosome polymorphic *Alu* insertions (PAIs)^47^, as well as direct sequencing^44^ as deemed necessary. The Y-SNP markers were scored in a hierarchal order to determine the Y-haplogroup status of each individual sample. Y-SNP haplogroup assignment and nomenclature is in accordance with the Y Chromosome Consortium^48^ and subsequent updates by Underhill et al.^49^. The S116 frequency reported represent total S116 as previously reported^41^.

### Y-STR haplotyping

The same samples genotyped for Y-SNPs were typed for 17 Y-STR loci (DYS19, DYS385a/b, DYS398I/II, DYS390, DYS391, DYS392, DYS393, DYS437, DYS438, DYS439, DYS448, DYS456, DYS458, DYS635, Y-GATA H4) using the AmpFlSTR Yfiler PCR amplification kit as recommended (Applied Biosystems of Life Technologies/Fisher Scientific). Resulting amplicons were separated on an ABI Prism 3130 XL Genetic Analyzer using the ABI GeneScan 500 LIZ as an internal size standard and fragment lengths were estimated by GeneMapper v3.2 (Thermo Scientific). Y-STR alleles were assigned by comparison with an allelic ladder provided by the manufacturer. The number of repeats at DYS389II was calculated after subtracting the number of repeats at DYS389I. Allelic nomenclature follows the recommendations of the International Society for Forensic Genetics (ISFG) (www.isfg.org).

### Accession numbers

Haplotypes for all the individuals of the Guipuzcoa, Vizcaya and Alava populations have been successfully submitted and are now included in the YHRD database under the following accession numbers: Guipuzcoa YP000722; Vizcaya YP000721; Alava YP000720.

### Statistical phylogenetic analyses

The Y-SNP and Y-STR frequency distributions of the Alava, Guipuzcoa and Vizcaya populations were evaluated within the context of 30 geographically targeted reference populations (Supplementary Table 1) in order to assess trans-continental (North West Europe, Western Europe, Eastern Europe, North Africa and West Asia) Y-chromosomal distribution patterns. Not all reference populations are included in every Y-SNP or Y-STR analysis.

Allelic frequencies were calculated with the PowerMarker V3.25^50^ program. Haplotype and haplogroup diversities were computed using the software package Arlequin V3.5^51^. All phylogenetic analyses were performed using the loci in common among the collections listed in Supplementary Table 1. DYS385 was excluded from the haplotype diversity calculations because is not possible to discriminate between the DYS385a and DYS385b loci with the Y STR kit. The number of repeats at DYS389II was calculated after subtracting the number of repeats at DYS389I. Discrimination capacity was calculated by dividing the number of different haplotypes by the total number of individuals in the population. The fraction of unique haplotypes was determined as the percent proportion of unique haplotypes.

Population pairwise genetic distances (*Rst* values) and the corresponding *P*-values were calculated for all given pairs of populations using the Arlequin v3.5 software^51^. The pairwise population comparisons were tested at a significance level of 0.01 with 10,000 permutations^52^. In order to compensate for potential inclusion of false positives, type I statistical errors, the Bonferroni correction was applied (α/m = 0.01/528 = 1.89394 × 10^–5^). The DYS385 locus was not included in population comparison for the reasons previously stated. All samples carrying non-consensus alleles and null alleles were excluded from the *Rst* calculations. Subsequently, multidimensional scaling (MDS) plots were generated in order to examine the phylogenetic relationships among populations in Supplementary Table 1. The MDS plot was constructed with SPSS v14.0^53^ using the *Rst* pairwise values. A Neighbor Joining (NJ) tree, based on *Fst* distances^54^, was constructed with the software PHYLIP 3.52c^55^ in order to deduce phylogenetic relationships between the populations under analysis. Bootstrap analysis involved 1000 replications.

Y-STR haplotypes of individuals belonging to the R-S116 haplogroup were used to generate a Median-Joining networks (NETWORK 4.5.1.6 at http://www.fluxusengineering), in which the Y-STR markers were weighted inversely to their repeat variance and the Maximum Parsimony (MP) option was employed to produce the least complex topology.

Y-STR haplotypes were used to estimate the time to the most recent common ancestor (TMRCA) of the R-S116 sub-haplogroup. With this aim, rho statistic (*ρ*)^56^ and weighted rho (*ρ*_W_)^12^ were estimated with an R script available in GitHub (http://github.com/fcalafell/weighted_rho). The number of repeats at DYS389II was calculated after subtracting the number of repeats at DYS389I. Mutation rates were obtained from the Y-Chromosome STR Haplotype Database (YHRD, www.yhrd.org) on March, 2020. The statistical significance of the time estimate differences were assessed using the Past 4.02 software (http://palaeo-electronica.org/2001_1/past/issue1_01.htm).

### Ethical standards

The IRB of Colorado College approved this study. All experimental protocols were approved by the IRB of Colorado College.

## Results

### Allelic frequencies and haplotypes

Supplementary Tables 2, 3 and 4 provide the allelic frequencies of the 17 Y-STR loci for the autochthonous Basque groups from Alava, Guipuzcoa and Vizcaya, respectively. Supplementary Tables 5, 6 and 7 provide the 17-loci haplotypes of 54, 30 and 61 individuals from Alava, Guipuzcoa and Vizcaya, respectively. The modal haplotypes of Alava, Guipuzcoa and Vizcaya are provided in Supplementary Table 8. Both Guipuzcoa and Vizcaya exhibit identical modal haplotypes while Alava differs only at DYS456 by one mutational step. For comparison the partial Irish^57^ modal haplotype is included in Supplementary Table 8. An Atlantic modal haplotype has been reported by Wilson and colleagues based on the Basque, Irish and Welsh populations^58^. Since not all Y-STR loci genotyped for the Basque populations in the present study were previously genotyped in the Irish population, not all the corresponding markers are available in the Irish modal haplotype. Of the ten comparable loci between the three Basque populations and the Irish, two differ by one mutational step.

### Forensic and population genetic parameters

Table 1 provides the forensic and population genetics parameters of Alava, Guipuzcoa and Vizcaya populations using the Minimal 9-loci, Extended 11-loci and Y-filer 17-loci haplotypes. As expected the values for number of haplotypes, unique haplotypes, fraction of unique haplotypes, discrimination capacity and haplotype diversity values augmented as the number of loci increased. Using the 17 loci included in the Y-filer system, the Alava, Guipuzcoa and Vizcaya populations exhibit high levels of genetic diversity, with haplotype diversity values of 1.0000, 0.9978 and 0.9978 as well as fraction of unique haplotypes of 1.00, 0.89 and 0.92, respectively. Discrimination capacity values of 0.94, 1.00 and 0.95 were estimated for Alava, Guipuzcoa, and Vizcaya, respectively. In general the increment to the 17-loci level of the Yfiler system as compared to the 9-loci of the Minimal and 11-loci Extended systems improves the resolution of discrimination in all three populations, especially for the Guipuzcoa group, which possesses fewer individuals. Non-consensus alleles were confirmed by repeating the amplification process.

**Table 1:** Forensic and population genetics parameters of the Alava, Guipuzcoa and Vizcaya Basque populations using the minimal, extended and the Yfiler haplotypes.

Haplotypes	Alava	Guipuzcoa	Vizcaya	All populations
**Minimal 9-loci Y-STR haplotype**				
Sample size	54	30	61	144
Number of haplotypes	38	22	40	84
Unique haplotypes	29	16	31	61
Fraction of unique haplotypes	0.55	0.53	0.51	0.42
Discrimination capacity	0.72	0.73	0.66	0.58
Haplotype diversity ± SD	0.9797 ± 0.0099	0.9770 ± 0.0145	0.9683 ± 0.0127	0.9746 ± 0.0068
**Extended 11-loci Y-STR haplotype**				
Sample size	54	30	61	144
Number of haplotypes	41	25	45	94
Unique haplotypes	32	21	36	69
Fraction of unique haplotypes	0.60	0.70	0.59	0.48
Discrimination capacity	0.77	0.83	0.74	0.65
Haplotype diversity ± SD	0.9891 ± 0.0060	0.9862 ± 0.0129	0.9836 ± 0.0078	0.9869 ± 0.0039
**Y-filer 17-loci Y-STR haplotype**				
Sample size	54	30	61	144
Number of haplotypes	50	30	58	127
Unique haplotypes	47	30	56	112
Fraction of unique haplotypes	0.89	1.00	0.92	0.78
Discrimination capacity	0.94	1.00	0.95	0.88
Haplotype diversity ± SD	0.9978 ± 0.0042	1.0000 ± 0.0086	0.9978 ± 0.0037	0.9982 ± 0.0011

### Phylogenetic analyses

#### Y-SNP haplogroups

Y-SNP haplogroups and their frequencies are shown in Supplementary Table 9. Six major haplogroups (R, I, E, J, G, and DE) were detected in 145 individuals from three Spanish Basque provinces of Alava (n = 54), Guipuzcoa (n = 30) and Vizcaya (n = 61), haplogroup R-S116 being the most abundant, ranging from 87.3% in Vizcaya and 86.7% in Guipuzcoa to 75.0% in Alava. None of the frequency differences by pairs of populations were statistically significant (Vizcaya-Alava: ts [test statistic] = 1.704, p = 0.088; Vizcaya-Guipuzcoa: ts = 0.080, p = 0.936; Guipuzcoa-Alava: ts = 1.320, p = 0.187). In the rest of the Iberian Peninsula the abundance of R-S116 ranges from 43% in Malaga to 81% in Catalonia while in other Western European populations, where its frequency is high, its prevalence is 80.69%, 74.66%, 50.91% and 16.67% in Brittany (France), Ireland, Portugal and Denmark, respectively^40^. The only other haplogroup that exhibits frequencies in double digits in two of the three Basque provinces examined is E-V65 (Alava, 17.3% and Vizcaya, 10.9%). E-V65 is of Northern African origin and could have dispersed into Iberia across the Mediterranean at various times since its origin approximately 4300 years ago. All the other haplogroups range from 0 to 6.7% depending on the Basque population. J-L24 and G-M287 are markers associated with the agricultural/Neolithic revolution while I-M253 is linked to Norse dispersals of the late eighth to late eleventh centuries of the current era. When the full set of haplogroups are considered together among the Basque populations, the observed differences are not statistical significant (Exact *P* value = 0.07814 + − 0.00970).

#### Y-STR haplotypes

Pairwise *Rst* genetic distances and the corresponding *P* values among Alava, Guipuzcoa and Vizcaya and the 30 geographically targeted reference populations are presented in Supplementary Table 10. The lowest *Rst* distances were detected among Alava, Guipuzcoa and Vizcaya populations. Comparisons between Alava, Guipuzcoa and Vizcaya, and the reference populations generated close *Rst* distances in relation to Basque Country, Gascony, Ireland, USA Basques, Andalusia and Spain (Supplementary Table 1), the lower distances in the order listed. Subsequent to the application of the Bonferroni adjustment for potential type I errors (α/m = 0.01/528 = 1.89394 × 10^–5^), additional pairwise *Rst* genetic distances were found to be statistically insignificant.

**Figure 2:**
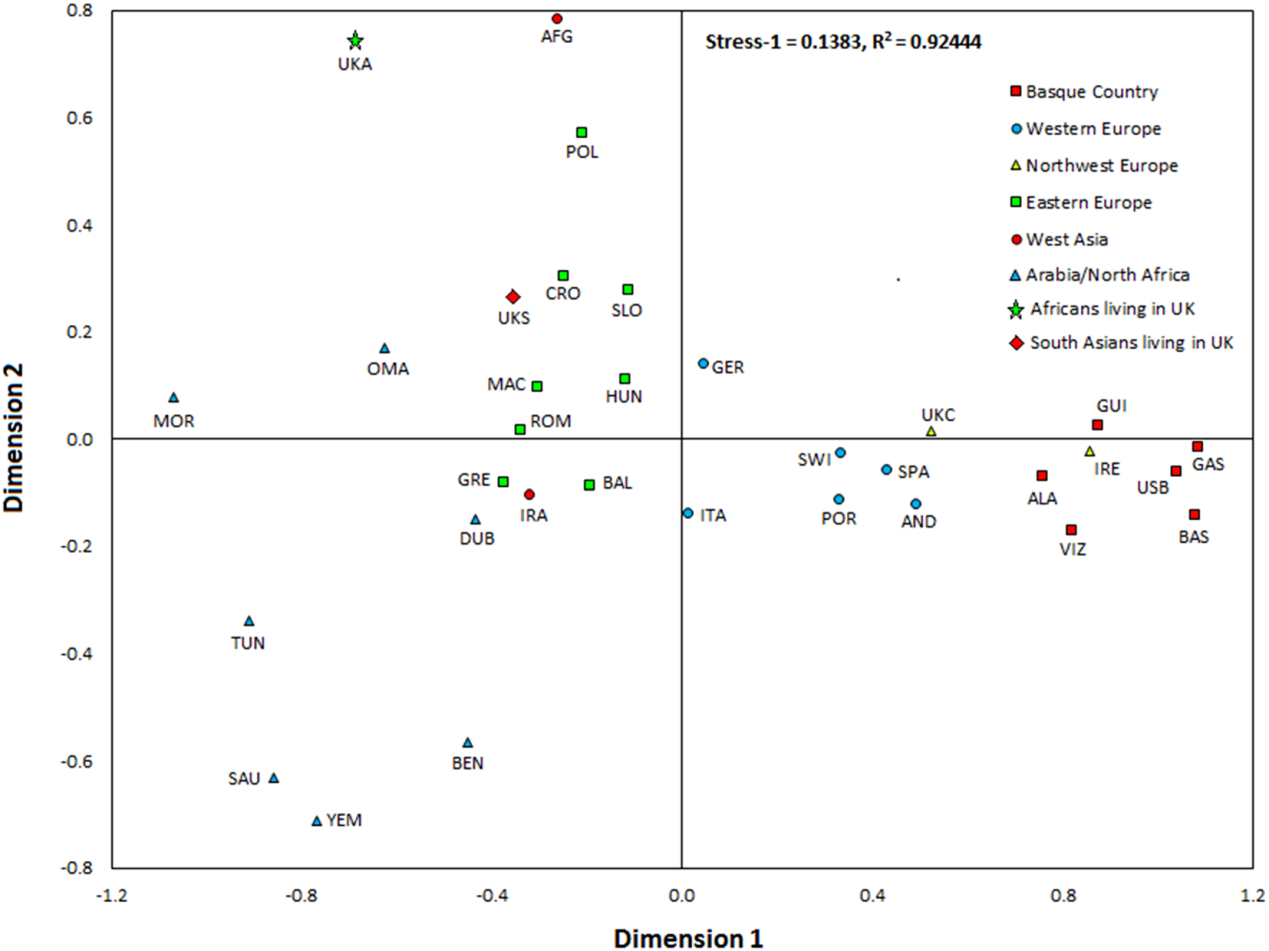
MDS plot based on *Rst* distances. Stress value = 0.1383, R^2^ = 0.92444. SPSS v14.0 at http://www.qlucore.com/visualize_data.

**Figure 3:**
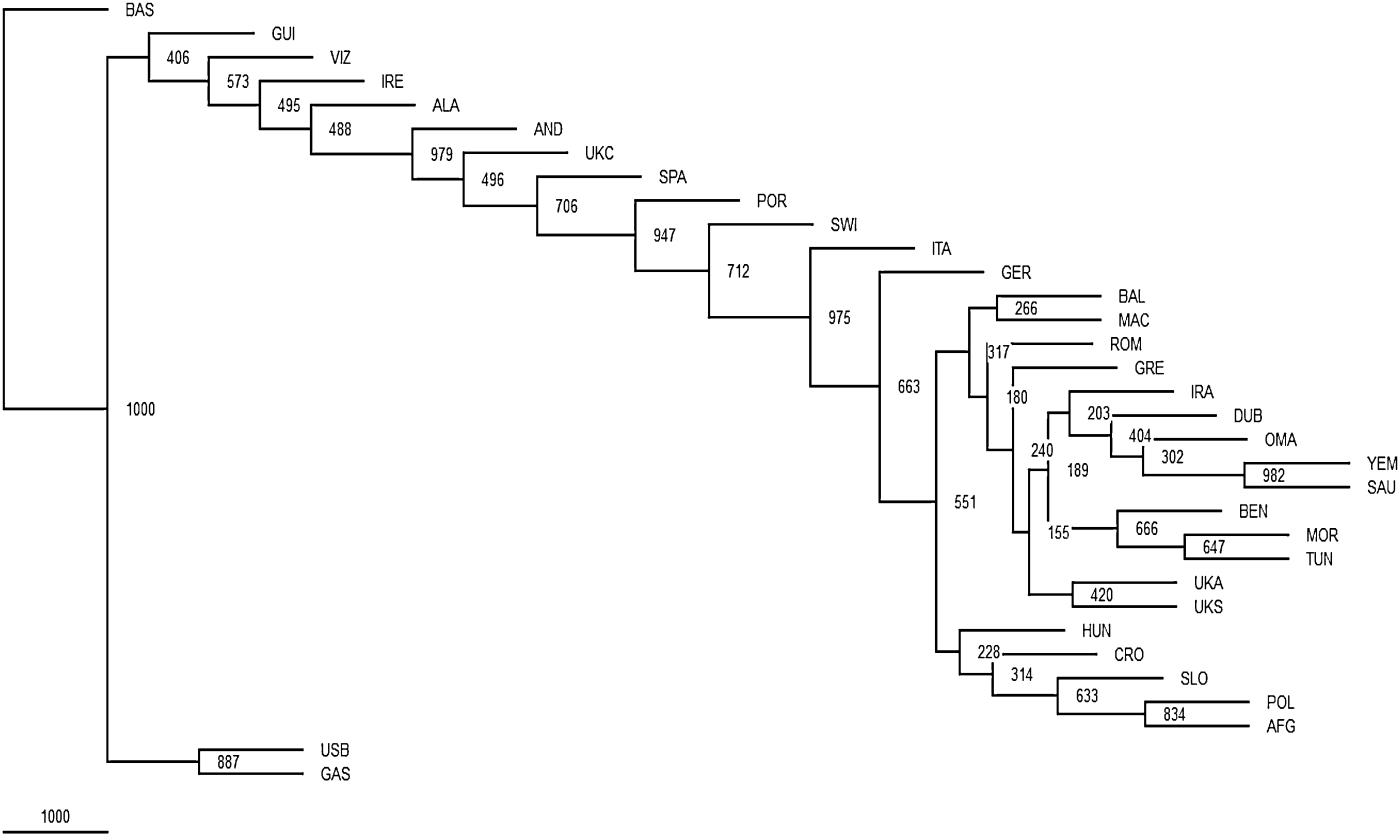
NJ tree. PHYLIP 3.52c at https://www.researchgate.net.

The Phylogenetic relationships among the three autochthonous Basque populations under study and the 30 geographically targeted key reference populations were evaluated using MDS analysis based on the *Rst* distances (Fig. 2). In the MDS graph, the populations from Alava, Guipuzcoa and Vizcaya segregate together in a cluster with the reference groups from the Basque Country (general population), Gascony, Ireland and the USA Basques to the right-center of the plot. Further to the left along the X-axis, the rest of the Western European populations form a cluster. The Eastern European reference populations follow to the left along the X-axis. The West Asian, Arabian and North African groups partition widely along the Y-axis at the far left of the graph. The groups of Africa and South Asian ancestry living in the United Kingdom are disperse within this last loose cluster. Within the European populations, the partitioning in the plot mirrors their west to east geographical distribution. The NJ projection based on different algorithms (Fig. 3) illustrates the same general topology for the European populations as the MDS graph. The cluster made up of Alava, Guipuzcoa, Vizcaya and the reference groups from the Basque Country, Gascony, Ireland and the USA Basque in the MDS plot (Fig. 2) also partition in proximity in the NJ tree with bootstrap values ranging from 100 to 40.6%. Of the populations in this Basque-Irish assembly, Alava segregates closer to non-Basque Western European populations. Discrete groupings of the Balkan region (Balkan, Macedonia, Romania and Greece), North African and Arabian populations are evident in the NJ tree. Yet, it is not clear why the United Kingdom populations of South Asian (UKS) and African (UKA) descend partition together (bootstrap = 42%), since in the MDS plot these two populations do not segregate in close proximity.

### Population structure and age estimates of haplogroup R-S116

**Figure 4:**
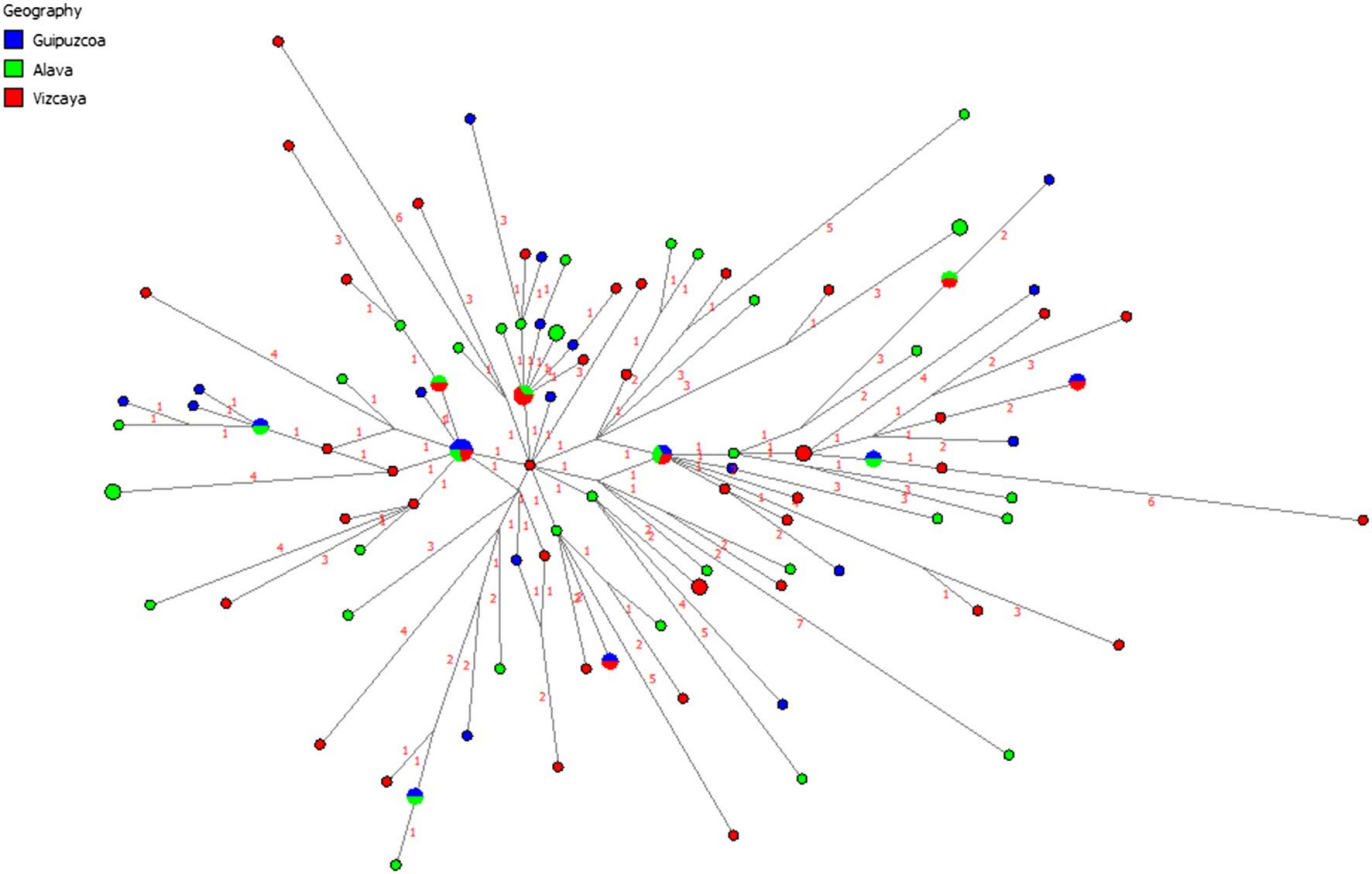
R1b-116 Network analysis. NETWORK 4.5.1.6 at http://www.fluxusengineering.

Considering the haplogroup homogeneity of the autochthonous Basque populations examined in this study, network analysis was performed to assess the genetic structure within haplogroup R-S116 (Fig. 4). The network was generated from the Y-STR haplotype data (DYS19, DYS389I/II, DYS390, DYS391, DYS392, DYS393, DYS437, DYS438, DYS439, DYS448, DYS456, DYS458, DYS635, Y-GATA H4) of the Alava, Guipuzcoa and Vizcaya populations (Supplementary Tables 5, 6 and 7). The network exhibits a symmetrical star-shape topology centered in an individual from Vizcaya. Most of the individuals are singletons or doubletons and there are only ten inter-population haplotype nodes, eight of which involve Alava and only two include the three autochthonous Basque populations. The network does not exhibit intra- or inter-population substructure. The population-specific haplotypes are dispersed uniformly throughout the plot. Often individuals from different populations emanate from a different population-specific node. The vast majority of individuals are separated by one-step mutation event.

The oldest age for haplogroup R-S116 (Supplementary Table 11) is found in the Vizcaya population at 4553 ± 285 years (weighted age, 30 years/generation). Alava and Guipuzcoa exhibit younger time estimates at 3975 ± 303 and 3680 ± 345 years (weighted age, 30 years/generation), respectively. Ages calculated based on 25 years per generation generate more recent ages (Supplementary Table 11). All age estimate comparisons among Alava, Guipuzcoa and Vizcaya were found to be statistically insignificant.

## Discussion

Examination of the Y haplogroups present in the autochthonous Basque populations of Alava, Guipuzcoa, Vizcaya indicate qualitative and quantitative differences among the three. Yet, these differences were not statistically significant. The R-S116 haplogroup predominates in all three populations, although it is less abundant in the population of Alava (Supplementary Table 9). The other haplogroups present such as I-M253, J-L24 and G-P287 are minor contributors and differ in frequencies in the three autochthonous Basque groups. Noteworthy, the Neolithic markers J-L24 and G-P287 were detected only at frequencies of 3.8% and 3.3% in Alava and Guipuzcoa, respectively. No Neolithic haplogroups were found in Vizcaya. No Paleolithic Y chromosomes were observed in the three Basque populations^59^.

The observed abundance of R-S116 in the autochthonous Basque populations is of particular interest. At 87.3% and 86.7% frequencies in Vizcaya and Guipuzcoa, respectively, these two Basques groups represent the highest recorded worldwide^40^. The R-S116 mutation and its ancestor polymorphism R-M269 have been associated with the spread of Indo-European languages in Western Europe during the Bronze Age^60,61^. The R-S116 mutation likely originated in what is today France about 5500–5000 years ago from a R-M269 background and subsequently spread to Iberia and Northwestern Europe^41^. Resulting from these Indo-European migrations that originated in the Pontic-Caspian steppe, it is theorized that these western European populations experienced a patrilineal genetic replacement from a Paleolithic/Neolithic composition to a Bronze Age Y-chromosome composition. However, this Bronze Age replacement disproportionally affected the Y-chromosome since a lot of the mtDNA in Iberia is still of Paleolithic origin (haplogroups H1, H3, U5 or V)^62^ and autosomal DNA is not uniform in Western Europe^62^. It is not clear which evolutionary force(s) were at play to account for this sex-bias replacement. It has been proposed that the invading Bronze Age populations carrying the R-S116 mutation possessed superior technology including bronze weapons, horses and wheeled vehicles, thus could have easily subjugated and/or annihilated most of the native Neolithic farmers and any remaining hunter-gatherers. Culturally, these invaders were warlike and priced heroism and conquest^63^. Another potential factor that may explain the observed Y-chromosome bias is that invading armies are mainly made-up of men and casualties are usually inflicted on the male population leaving the woman for the winners to reproduce.

The age estimates of R-S116 are congruent with its origin somewhere in France approximately 5500–5000 years ago^41^ considering a migration to the Basque Country of a population carrying it. The TMRCA for the Spaninsh Basque population as a whole for haplogroups I2 and J2a have been reported at 7800 ya and 5500 ya, both older than R-S116^12^. These older ages for I2 and J2a in the Basque are expected considering that these haplogroups arrived in Europe during the Neolithic. Yet, similar expansion times (about 50 generations ago or 1200 ya) and estimated initial and final populations for haplogroups R-S116, G2a, I2, and J2a in the Spanish Basque as a whole have been reported^12^. It is not known if the same demographic forces affected the whole of the Y chromosome composution in other parts of Iberia.

As a result of the shortcomings related to the estimation of Y-STR mutation rates, the ages provided in this article should only be consider as relative assessments. As indicated by Ballantyne et al.^64^ and Busby et al.^65^, mutation rates vary considerable among Y-STR loci and alleles, therefore, correspondence involving studies using different STR loci are difficult. Furthermore, age estimations computed using Y-STR diversity are affected by various factors that may overestimate the values, including several incursions from different ancestral populations. Therefore, age estimates should be considered as upper bounds. Notwithstanding these considerations, the ages reported in the present study are suitable for comparisons involving the populations analyzed in this study.

A review of the *Rst* values (Supplementary Table 10) demonstrates that the populations of Alava, Guipuzcoa, Vizcaya are genetically closer to each other than to any of the reference populations, being the *Rst* values statistically insignificant. Notable, the genetic distances separating Ireland from the Alava, Guipuzcoa, Vizcaya and United State Basques populations became statistically insignificant when the Bonferroni correction was applied. Also, the genetic distances of Gascony to the Alava, Guipuzcoa, Vizcaya, general Basques and United State Basques were statistical insignificant even prior to the Bonferroni correction. Considering the distant geographic location of Ireland in Northern Europe to the Basque Provinces, these genetic affinities suggest some degree of common ancestry among these populations, likely related to the homogenizing effect of the Bronze Age dispersals also reflected in the high levels of the R-S116 mutation throughout the region.

The MDS illustrates a longitudinal partitioning of populations with the Basque groups at one end and more geographically easterly groups progressively to the left of the plot. The population of Ireland stands out from this geographically specific partitioning. Ireland segregates within the Basque cluster. In fact Ireland′s closest group in the MDS is the population of Guipuzcoa, the most geographically easterly of the Basque groups. Furthermore, in the MDS Guipuzcoa is closer to Ireland than to any of the other Basque populations. For comparison, the Caucasian population of the United Kingdom segregates with the non-Basque Western European cluster. Confirmation of these genetic affinities between the Irish and the Basque populations is seen in the topology of the NJ tree (Fig. 3) generated using different algorithms relative to the *Rst* and MDS analyses. In this discussion of the similarities between the Basques and Irish at the level of haplogroup R-S116, it is important to indicate that the formers exhibit high frequencies of R-DF27 and the later of R-M529^12,42^ suggesting that these mutations may have occurred in situ in the respective regions on R-S116 individuals.

The resulting R-S116 network’s topology based on the three Spanish Basque populations is star-shaped and symmetrical (Fig. 4). Most of the nodes are singletons or doubletons and only ten are shared among the three Basque populations. Three of the haplotypes (nodes) shared among the populations are located near the center of the network and emanating from them are singleton haplotypes of individuals residing in the three provinces. Also, most of the nodes are separated by only one or two mutation events. No inter-population structure is seen in the network as individuals from the three groups are randomly distributed throughout the network. This suggests a recent population expansion with the three autochthonous populations separating shortly after the arrival of the R-S116 migrants to the Basque region. Also, the random distribution of individuals belonging to the three Basque populations within the network indicates that people with similar haplotypes live in the three provinces. This is likely the result of substantial movement of individuals within the Basque Country. The random distribution of R-S116 haplotypes within the network argues for Y-chromosome homogeneity within the Basque Country.

The relationship of the Y-STR haplotypes of the three Basque populations under R-S116 to other European populations was examined by MDS and Network analyses. The partitioning pattern observed in the MDS plot based on Y-STR haplotype diversity under sub-haplogroup R-S116 (Supplementary Fig. 1) contrast dramatically with the partition of European population observed in the MDS projection based on the entire Y-STR haplotype diversity (Fig. 2). Populations such as Netherlands, Sweden, Hungary and Germany, among others, segregate as part of a cluster in the R-S116 plot as compared to a rather disperse partition of European populations in the general MDS. By restricting the analysis to diversity within R-S116, the genetic affinity among the European population has been reduced. Similarly, the Network analysis exhibits a random distribution of European populations into a number of inter-population star-like nodes separated by a small number of mutational steps (Supplementary Fig. 2). Notably, the larger nodes are made up of most of the populations analyzed. There is no evidence of inter- or intra-population structure. This outcome reflects limited Y-STR haplotype diversity within R-S116 among European populations suggesting a recent contemporaneous separation of European populations carrying the S116 mutation. This scenario is compatible with the hypothesis that a swift dispersal of Indo-European invaders from the STEPPES into Europe about four to five millennia ago led to Y chromosome replacement.

## Conclusion

The analyses performed in this investigation support the hypothesis that during the Bronze Age a dispersal of individuals occurred that led to the replacement of the Paleolithic/Neolithic Y-chromosome composition in Western Europe by Indo-European R-S116 lineages. Our data shows that this substitution was not uniform and that in some localities such as the Basque Country of Spain, the replacement was more vast and thorough than in other regions of Western Europe. Although our data does not uncover the evolutionary mechanism(s) that brought about such a specific and dramatic replacement of Y-chromosome types, it demonstrates that the Bronze Age dispersal genetically linked populations as geographically dispersed as Ireland, Gascony and the Basque Country of Spain.

## Supplementary Information

Supplementary Information 1.

Supplementary Figure 1.

Supplementary Figure 2.

Supplementary Table 1.

Supplementary Table 2.

Supplementary Table 3.

Supplementary Table 4.

Supplementary Table 5.

Supplementary Table 6.

Supplementary Table 7.

Supplementary Table 8.

Supplementary Table 9.

Supplementary Table 10.

Supplementary Table 11.

## References

[1] Cavalli-Sforza LL (1988). The Basque population and ancient migration in Europe. Munibe.

[2] Cavalli-Sforza LL, Menozzi P, Piazza A (1994). The History and Geography of Human Genes.

[3] Calafell F, Bertranpetit J (1994). Principal component analysis of gene frequencies and the origin of Basques. Am. J. Phys. Anthropol..

[4] Calafell F, Bertranpetit J (1994). Mountains and genes: Population history of the Pyrenees. Hum. Biol..

[5] Alonso S, Armour JA (1998). MS 205 minisatellite diversity in Basques: Evidence for a pre-Neolithic component. Genome Res..

[6] Zlojutro M, Roy R, Palikij J, Crawford MH (2006). Autosomal STR variation in a Basque population: Vizcaya Province. Hum. Biol..

[7] Garcia O, Martin P, Gusmao L, Albarran C, Alonso S, de la Rua C (2004). A Basque Country autochthonous population study of 11 Y-chromosome STR loci. Forensic Sci. Int..

[8] Valverde L, Köhnemann S, Rosique M, Cardoso S, Zarrabeitia M, Pfeiffer H, de Pancorbo MM (2012). 17 Y-STR haplotype data for a population sample of residents in the Basque Country. Forensic Sci. Int. Genet..

[9] Nuñez C (2015). Highly discriminatory capacity of the PowerPlex(®) Y23 System for the study of isolated populations. Forensic Sci. Int. Genet..

[10] Baeta M (2018). Assessment of a subset of Slowly Mutating Y-STRs for forensic and evolutionary studies. Forensic Sci. Int. Genet..

[11] Hallast P, Batini C, Zadik D, Maisano Delser P, Wetton JH, Arroyo-Pardo E (2015). The Y-chromosome tree bursts into leaf: 13,000 high-confidence SNPs covering the majority of known clades. Mol. Biol. Evol..

[12] Solé-Morata N (2017). Analysis of the R1b-DF27 haplogroup shows that a large fraction of Iberian Y-chromosome lineages originated recently in situ. Sci. Rep..

[13] Corte-Real HB, Macaubay VA, Richards MB, Hariti G, Issad MS, Cambon-Thosen A, Papiba S, Bertranpetit J, Sykes BC (1996). Genetic diversity in the Iberian Peninsula determined from mitochondrial sequence analysis. Ann. Hum. Genet..

[14] Martinez de Pancorbo M, López-Martínez M, Martínez-Bouzas C, Castro A, Fernández-Fernández I, Antunez de Mayolo G (2001). The Basques according to polymorphic *Alu* insertions. Hum. Genet..

[15] Günther T, Valdiosera C, Malmström H, Ureña I, Rodriguez-Varela R, Sverrisdóttir ÓO (2015). Ancient genomes link early farmers from Atapuerca in Spain to modern-day Basques. PNAS.

[16] Comas D, Matew E, Calafell F, Perez-Lezaun A, Bosch E, Martinez-Arias R, Bertranpetit J (1998). HLA class I and class II DNA typing and the origin of Basques. Tissue Antigens.

[17] Bertranpetit J, Sala J, Calafell F, Underhill PA, Moral P, Comas D (1995). Human mitochondrial DNA variation and the origin of Basques. Ann. Hum. Genet..

[18] Alzualde A, Izagirre N, Alonso S, Alonso A, de la Rua C (2005). Temporal mitochondrial DNA variation in the Basque country: Influence of post-Neolithic events. Ann. Hum. Genet..

[19] Arrieta MI, Martinez B, Millan JM, Gil A, Monros E, Nuñez T, Telez M, Martinez F (1997). Study of a trimeric tandem repeat locus (SBMA) in the Basque population: Comparison with other populations. Gene Geogr..

[20] Adams SM, Bosch E, Balaresque PL (2008). The genetic legacy of religious diversity and intolerance: Paternal lineages of Christians, Jews, and Muslims in the Iberian Peninsula. Am. J. Hum. Genet..

[21] Alonso S, Flores C, Cabrera V, Alonso A, Martin P, Albarran C, Izagirre N, de la Rua C, Garcia O (2005). The place of the Basques in the European Y-chromosome diversity landscape. Eur. J. Hum. Genet..

[22] Garagnani P, Laayouni H, Gonzalez-Neira A, Sikora M, Luiselli D, Bertranpetit J, Calafell F (2009). Isolated populations as treasure troves in genetic epidemiology: The case of the Basques. Eur. J. Hum. Genet..

[23] Laayouni H, Calafell F, Bertranpetit J (2010). A genome-wide survey does not show the genetic distinctiveness of Basques. Hum. Genet..

[24] Comas D, Calafell F, Mateu E, Perez-Lezaun A, Bertranpetit J (1998). HLA evidence for the lack of genetic heterogeneity in Basques. Ann. Hum. Genet..

[25] Rodriguez-Ezpeleta N, Alvarez-Busto J, Imaz L, Regueiro M, Azcarate MN, Bilbao R, Iriondo M, Gil A, Estonba A, Aransay AM (2010). High-density SNP genotyping detects homogeneity of Spanish and French Basques, and confirms their genomic distinctiveness from other European populations. Hum. Genet..

[26] Manzano C, de la Rua C, Iriondo M, Mazon LI, Vicario A, Aguirre A (2002). Structuring the genetic heterogeneity of the Basque population: A view from classical polymorphisms. Hum. Biol..

[27] Iriondo M, Barbero MC, Manzano C (2003). DNA polymorphisms detect ancient barriers to gene flow in Basques. Am. J. Phys. Anthropol..

[28] Perez-Miranda AM, Alfonso-Sanchez MA, Kalantar A, Garcia Obregon S, de Pancorbo MM, Pena JA, Herrera RJ (2005). Microsatellite data support subpopulation structuring among Basques. J. Hum. Genet..

[29] Alfonso-Sanchez MA, Cardoso S, Martinez-Bouzas C, Pena JA, Herrera RJ, Castro A, Fernandez-Fernandez I, De Pancorbo MM (2008). Mitochondrial DNA haplogroup diversity in Basques: A reassessment based on HVI and HVII polymorphisms. Am. J. Hum. Biol..

[30] Aguirre AI, Vicario A, Mazón LI, Estomba A, de Pancorbo MM, Arrieta-Pico V, Pérez Elortondo F, Lostao CM (1991). Are the Basque a single and unique population?. Am. J. Hum. Genet..

[31] Manzano C, Aguirre AI, Iriondo M, Martin M, Osaba L, de la Rua C (1996). Genetic polymorphisms of the Basques from Gipuzkoa: Genetic heterogeneity of the Basque population. Ann. Hum. Biol..

[32] Manzano C, Orue JM, de la Rua C (1996). The “Basqueness” of the Basques of Alava: A reappraisal from a multidisciplinary perspective. Am. J. Phys. Anthropol..

[33] Esteban E, Dugoujon JM, Guitard E, Senegas MT, Manzano C, de la Rua C, Valveny N, Moral P (1998). Genetic diversity in northern Spain (Basque Country and Cantabria): GM and KM variation related to demographic histories. Eur. J. Hum. Genet..

[34] Calderon R, Perez-Miranda A, Peña JA, Vidales C, Aresti U, Dugoujon JM (2000). The genetic position of the autochthonous subpopulation of nothern Navarre (Spain) in relaiton to other Basque subpopulations. A study based on GM and KMimmunoglobulin allotypes. Hum. Biol..

[35] Izagirre N, de la Rua C (1999). An mtDNA analysis in ancient Basque populations: Implications for haplogroup V as a marker for a major paleolithic expansion from southwestern Europe. Am. J. Hum. Genet..

[36] Martınez-Cruz B, Harmant C, Platt DE, Haak W, Manry J, Ramos-Luis E (2012). Evidence of pre-Roman tribal genetic structure in Basques from uniparentally inherited markers. Mol. Biol. Evol. Res..

[37] Olalde I, Mallick S, Nick Patterson N, Rohland N, Villalba-Mouco V, Silva M, Katharina D (2019). The genomic history of the Iberian Peninsula over the past 8000 years. Science.

[38] Ruiz Zapatero, G. *Protohistory of the Far West of Europe: From Neolithic to Roman Conquest*. (Almagro-Gorbea, M. ed) (Universidad de Burgos, Fundación Atapuerca, 2014)

[39] Maca-Meyer N, Sánchez-Velasco P, Flores C, Larruga JM, González AM, Oterino A, Leyva-Cobián F (2003). Y chromosome and mitochondrial DNA characterization of Pasiegos, a human isolate from Cantabria (Spain). Ann. Hum. Genet..

[40] Flores C (2004). Reduced genetic structure of the Iberian Peninsula revealed by Y-chromosome analysis: implications for population demography. Eur. J. Hum. Genet..

[41] Myres NM, Rootsi S, Lin AA (2011). A major Y-chromosome haplogroup R1b Holocene era founder effect in Central and Western Europe. Eur. J. Hum. Genet..

[42] Valverde L, Illescas M, Villaescusa P (2016). New clues to the evolutionary history of the main European paternal lineage M269: Dissection of the Y-SNP S116 in Atlantic Europe and Iberia. Eur. J. Hum. Genet..

[43] Batzer MA, Deininger PL (1991). A human-specific subfamily of *Alu* sequences. Genomics.

[44] Luis J, Rowold D, Regueiro M, Caeiro JL, Cinnioglu C, Roseman C (2004). The Levant versus the Horn of Africa: Evidence for bidirectional corridors of human migrations. Am. J. Hum. Genet..

[45] Martinez L, Reategui E, Fonseca L, Sierra-Montes J, Terreros M, Pereira-Simon S (2005). Superimposing polymorphism: The case of a point mutation within a polymorphic Alu insertion. Hum. Hered..

[46] Regueiro M, Cadenas A, Gayden T, Underhill P, Herrera RJ (2006). Iran: Tri-continental nexus for Y-chromosome driven migration. Hum. Hered..

[47] Hammer M, Horai S (1995). Y-chromosomal DNA variation and the peopling of Japan. Am. J. Hum. Genet..

[48] Karafet T, Mendez F, Meilerman M, Underhill P, Zegura S, Hammer M (2008). New binary polymorphisms reshape and increase resolution of the human Y-chromosomal haplogroup tree. Genome Res..

[49] Underhill PA, Myres N, Rootsi S, Metspalu M, Zhivotovsky L, King R (2010). Separating the post-glacial co-ancestry of European and Asian Y-chromosomes within haplogroup R1a. Eur. J. Hum. Genet..

[50] Liu K, Muse SV (2005). PowerMarker: An integrated analysis environment for genetic marker analysis. Bioinformatics.

[51] Excoffier L, Lischer HEL (2010). Arlequin suite ver 3.5: A new series of programs to perform population genetics analyses under Linux and Windows. Mol. Ecol. Res..

[52] Kayser M, Brauer S, Schadlich H, Prinz M, Batzer MA, Zimmerman PA (2003). Y chromosome STR haplotypes and the genetic structure of U.S. populations of African, European, and Hispanic ancestry. Genome Res..

[53] SPSS for windows, rel. 11.0.1. (SPSS Inc., 2006).

[54] Reynolds J, Weir BS, Cockerham CC (1983). Estimation of the coancestry coefficient: Basis for a short-term genetic distance. Genetics.

[55] Felsenstein, J. *Phylogeny Inference Package (PHYLIP), Version 3.6a3. Distributed by Author*. (Department of Genetics, University of Washington, 2002).

[56] Saillard J, Forster P, Lynnerup N, Bandelt HJ, Nørby S (2000). mtDNA variation among Greenland Eskimos: The edge of the Beringian expansion. Am. J. Hum. Genet..

[57] Moore T (2006). A Y-chromosome signature of hegemony in Gaelic Ireland Laoise. Am. J. Hum. Genet..

[58] Wilson JF (2001). Genetic evidence for different male and female roles during cultural transitions in the British Isles. PNAS.

[59] Martinez L, Underhill PA, Zhivotovsky LA, Gayden T, Moschonas NM, Chow CET, Simon Conti S (2007). Paleolithic Y-haplogroup heritage predominates in a Cretan highland plateau. EJHG.

[60] Olalde I, Brace S, Allentoft ME, Armit I, Kristiansen K, Booth T (2018). The Beaker phenomenon and the genomic transformation of northwest Europe. Nature.

[61] Fu Q, Posth C, Hajdinjak M, Petr M, Mallick S, Fernandes D (2016). The genetic history of Ice Age Europe. Nature.

[62] Sampietro ML, Lao O, Caramelli D, Lari M, Pou R, Marti M, Bertranpetit J, Lalueza-Fox C (2007). Palaeogenetic evidence supports a dual modelof Neolithic spreading into Europe. Proc. R. Soc. B.

[63] Anthony DW (2007). The Horse, the Wheel, and Language: How Bronze-Age Riders from the Eurasian Steppes Shaped the Modern World.

[64] Ballantyne KN, Goedbloed M, Fang R, Schaap O, Lao O, Wollstein A (2010). Mutability of Y-chromosomal microsatellites: Rates, characteristics, molecular bases, and forensic implications. Am. J. Hum. Genet..

[65] Busby GB, Brisighelli F, Sanchez-Diz P, Ramos-Luis E, Martinez-Cadenas C, Thomas MG (2012). The peopling of Europe and the cautionary tale of Y chromosome lineage R-M269. Proc. Biol. Sci..

