# Supplementary material for: The Y chromosome of autochthonous Basque populations and the Bronze Age replacement
Source: Sci Rep. 2021 Mar 10;11:5607. doi: 10.1038/s41598-021-84915-1 (PMC7970938; doi:10.1038/s41598-021-84915-1)
Supplement: Supplementary file 1 — Supplementary Information 1. [file 41598_2021_84915_MOESM1_ESM.docx]

Human Subject Statement

All samples were procured from donors voluntarily while closely adhering to the ethical guidelines stipulated by Colorado College, Colorado Springs, Colorado USA. All donors gave their informed consent prior to inclusion in the study, following the ethical principles and guidelines of the Declaration of Helsinki for the protection of human subjects. The IRB of Colorado College approved this study. All experimental protocols were approved by the IRB of Colorado College
