# Supplementary figures and images for: The Y chromosome of autochthonous Basque populations and the Bronze Age replacement
Source: Sci Rep. 2021 Mar 10;11:5607. doi: 10.1038/s41598-021-84915-1 (PMC7970938; doi:10.1038/s41598-021-84915-1)

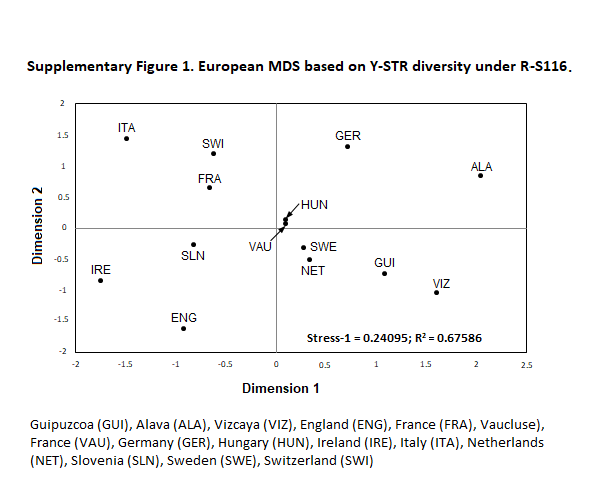

Supplement: Supplementary file 2 — Supplementary Figure 1. [file 41598_2021_84915_MOESM2_ESM.tif]

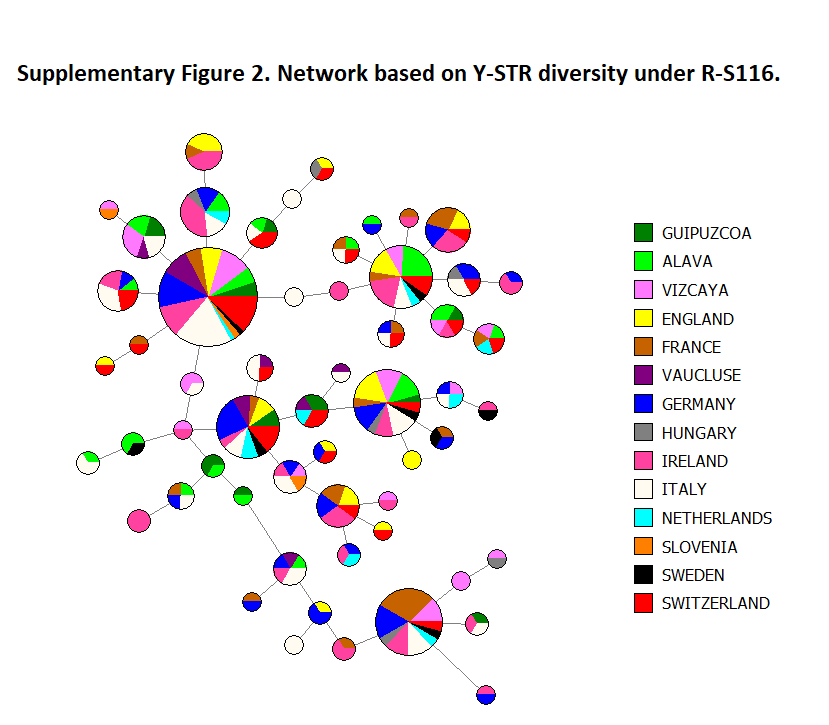

Supplement: Supplementary file 3 — Supplementary Figure 2. [file 41598_2021_84915_MOESM3_ESM.tif]

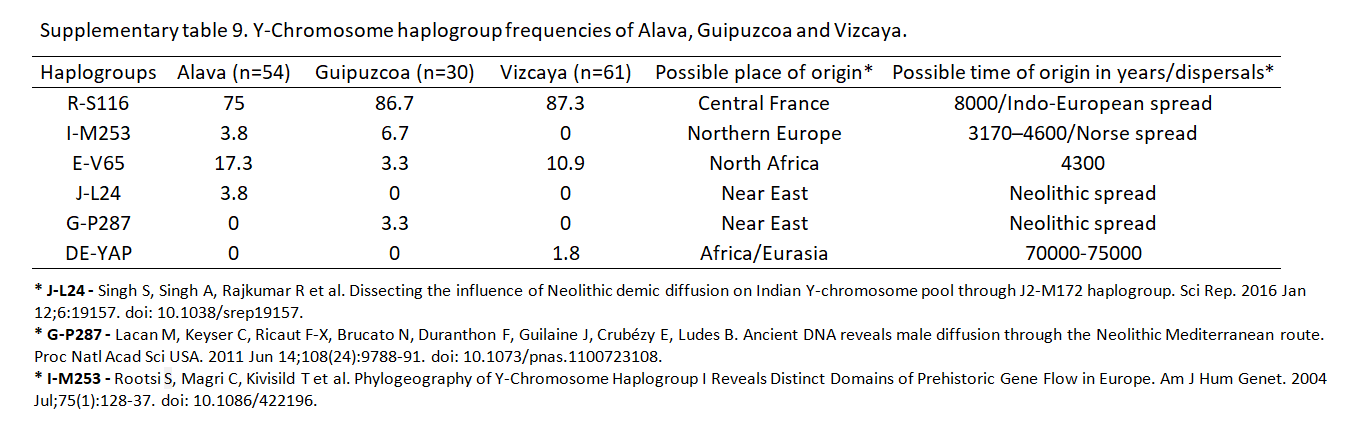

Supplement: Supplementary file 12 — Supplementary Table 9. [file 41598_2021_84915_MOESM12_ESM.tif]
